# PDE8 Regulates Rapid Teff Cell Adhesion and Proliferation Independent of ICER

**DOI:** 10.1371/journal.pone.0012011

**Published:** 2010-08-09

**Authors:** Amanda G. Vang, Shlomo Z. Ben-Sasson, Hongli Dong, Barbara Kream, Michael P. DeNinno, Michelle M. Claffey, William Housley, Robert B. Clark, Paul M. Epstein, Stefan Brocke

**Affiliations:** 1 Department of Immunology, University of Connecticut Health Center, Farmington, Connecticut, United States of America; 2 Department of Pharmacology, University of Connecticut Health Center, Farmington, Connecticut, United States of America; 3 Laboratory of Immunology, National Institute of Allergy and Infectious Diseases, National Institutes of Health, Bethesda, Maryland, United States of America; 4 Department of Cell Biology, University of Connecticut Health Center, Farmington, Connecticut, United States of America; 5 Department of Medicine, University of Connecticut Health Center, Farmington, Connecticut, United States of America; 6 Department of Genetics and Developmental Biology, University of Connecticut Health Center, Farmington, Connecticut, United States of America; 7 Pfizer Global Research and Development, Groton Laboratories, Groton, Connecticut, United States of America; New York University, United States of America

## Abstract

**Background:**

Abolishing the inhibitory signal of intracellular cAMP by phosphodiesterases (PDEs) is a prerequisite for effector T (Teff) cell function. While PDE4 plays a prominent role, its control of cAMP levels in Teff cells is not exclusive. T cell activation has been shown to induce PDE8, a PDE isoform with 40- to 100-fold greater affinity for cAMP than PDE4. Thus, we postulated that PDE8 is an important regulator of Teff cell functions.

**Methodology/Principal Findings:**

We found that Teff cells express PDE8 *in vivo*. Inhibition of PDE8 by the PDE inhibitor dipyridamole (DP) activates cAMP signaling and suppresses two major integrins involved in Teff cell adhesion. Accordingly, DP as well as the novel PDE8-selective inhibitor PF-4957325-00 suppress firm attachment of Teff cells to endothelial cells. Analysis of downstream signaling shows that DP suppresses proliferation and cytokine expression of Teff cells from *Crem* ^−/−^ mice lacking the inducible cAMP early repressor (ICER). Importantly, endothelial cells also express PDE8. DP treatment decreases vascular adhesion molecule and chemokine expression, while upregulating the tight junction molecule claudin-5. *In vivo*, DP reduces CXCL12 gene expression as determined by *in situ* probing of the mouse microvasculature by cell-selective laser-capture microdissection.

**Conclusion/Significance:**

Collectively, our data identify PDE8 as a novel target for suppression of Teff cell functions, including adhesion to endothelial cells.

## Introduction

The cyclic nucleotide phosphodiesterase (PDE)4 acts as a critical regulator of T cell function through its ability to hydrolyze intracellular cAMP [1]–[3]. However, substantial evidence supports the existence of PDE4-independent mechanisms of cAMP degradation in T cells [4]–[7]. In PDE4A^−/−^ and D^−/−^ mice, for example, T cell function is normal while in PDE4B^−/−^ mice, there is a more pronounced defect in macrophage function than in T cell proliferation [7]. Similarly, the PDE4-selective inhibitor rolipram only weakly suppresses proliferation of polyclonal T cell populations [8], [9] despite its effectiveness in selected T cell clones [10]. Additional analyses indicate that PDE4 accounts for less than 50% of total PDE activity in T cells [9]. Subsequently, PDEs other than PDE4 have been identified in T cells, and the overall PDE activity in T cells *in vitro* has now been attributed to PDE1, 2, 3, 4, 7 and 8 [4]–[6], [11]. Whether these different PDE activities identified *in vitro* operate *in vivo* remains an active field of investigation.

cAMP is a potent regulator of the immune response, mainly through activation of cAMP-dependent protein kinase A (PKA) and its established inhibitory action on effector T (Teff) cells [1], [9], [12]–[14]. Activation of receptors coupled to Gs proteins by extracellular ligands such as catecholamines, prostaglandins and adenosine causes accumulation of intracellular cAMP and leads to immunosuppression *in vivo* and *in vitro* [14]–[16]. Due to the detailed functional characterization of individual PDEs within the 11 member gene family, it is now accepted that distinct PDE isoforms regulate specific cell functions [2], [17]. These properties afford the opportunity to selectively inhibit PDE isoforms to treat defined pathologic conditions. Thus, the PDE superfamily emerged as a new target for the development of specific therapeutic agents [11], [18].

Notably, rolipram blocks experimental inflammation in animal models when applied before or during immunization [8], [19]. In contrast, its therapeutic efficacy is highly variable when treatment is initiated after the appearance of clinical signs [8], [19]–[21]. In clinical trials, pharmacological inhibitors of PDE4 developed as potential therapies for treatment of inflammatory diseases were less efficacious than preclinical data suggested [18], [21], [22]; consequently, none has yet been approved for clinical use [23], [24]. Consistent with these observations, recent studies indicated that the high affinity isoforms PDE7A and PDE8A are required for full T cell activation [5], [6].

These puzzling findings led us to question some of the prevailing assumptions regarding the mechanism of PDE control of cAMP signaling in T cells, and prompted us to investigate PDE expression in activated CD4^+^ T cells *in vivo* and the role of distinct members of the PDE superfamily in CD4^+^ T cell functions. The ability of T cells to firmly arrest on vascular endothelial cells and subsequently migrate into the target tissue through the endothelium is a key checkpoint during inflammatory lesion formation. We recently identified PDE8 as a novel target for inhibition of T cell chemotaxis [25]. However, unlike motility and chemotaxis in interstitial spaces, T cell interaction with vascular endothelium must maintain persistent resistance to detachment by disruptive shear forces of the blood flow [26], [27]. In activated T cells, three major integrins, LFA-1 (αLβ2) and the α4 integrins VLA-4 and α4β7, control essentially all shear-resistant interactions with endothelial cells.

Since the cAMP-PKA signaling pathway controls Teff cell adhesion to vascular ligands and regulates vascular barrier functions [28]–[31], we tested here the hypothesis that PDE8 – through hydrolysis of intracellular cAMP – may be an important regulator of T cell adhesion and thereby serve as a target for the inhibition of T cell recruitment to vascular endothelium. We now show that PDE8A is expressed in activated T cells *in vivo*. Mechanistic studies demonstrate that inhibiting PDE8 (i) is critical in rapid suppression of α4 and αL integrin expression and inhibition of T cell-endothelial cell interaction *in vitro*, (ii) decreases vascular adhesion molecule and chemokine expression and enhances expression of the tight junction molecule claudin-5 on endothelial cells *in vitro* and *in vivo*, and (iii) plays a significant role in the inhibition of proliferation and T helper-type 1 (Th1) cytokine production of CD4^+^CD25^−^ Teff cells through a cAMP-dependent but inducible cAMP early repressor (ICER)-independent mechanism. These data identify a non-redundant role for PDE8 in controlling T cell functions and have implications for the development of anti-inflammatory therapies based on targeting PDEs and activating cAMP signaling.

## Results

### Activated CD4+ Teff cells express PDE8A *in vivo* and *in vitro*

**Figure 1:**
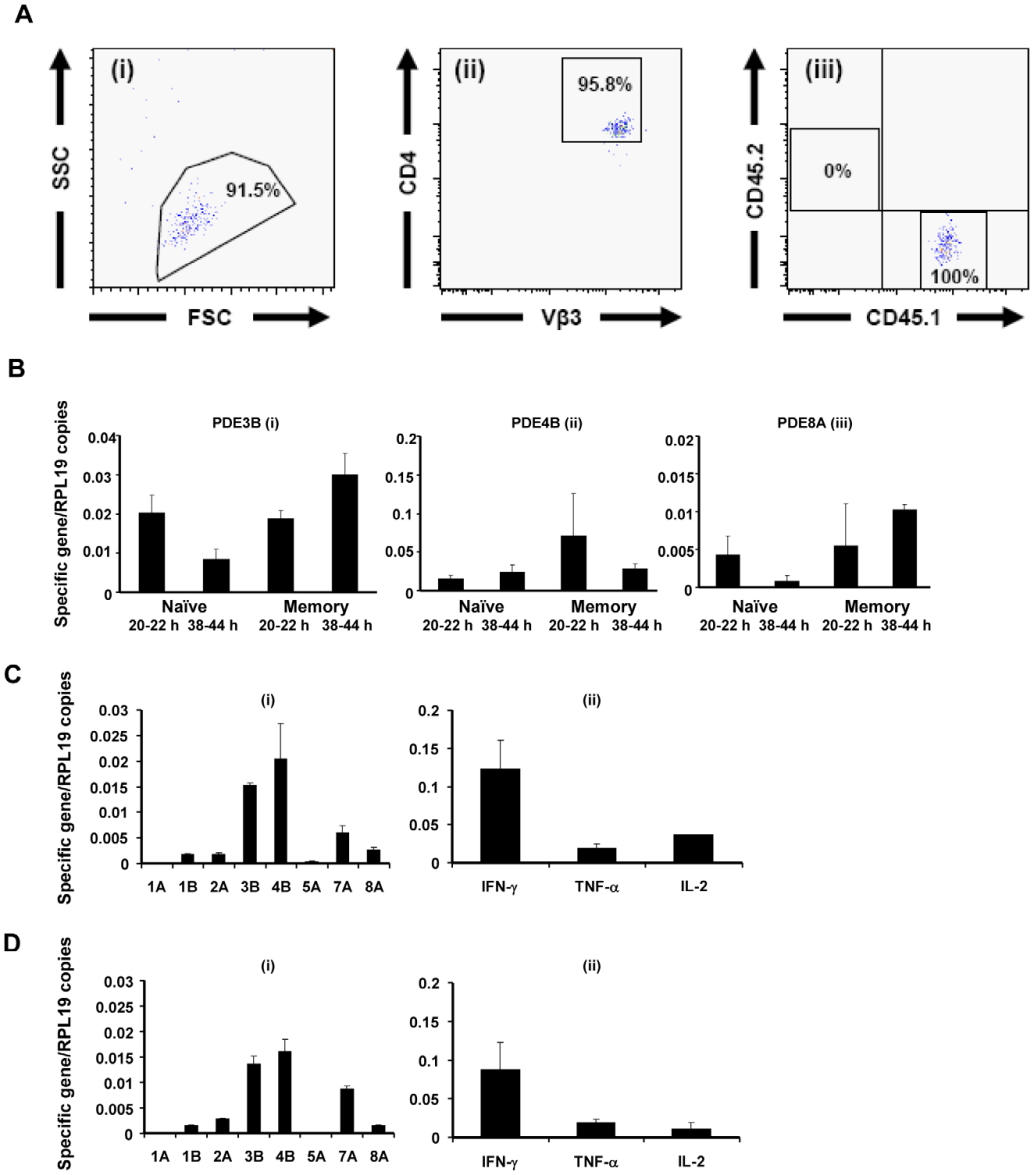
PDE8 is expressed in activated CD4^+^ T cells *in vivo* and *in vitro*. (A–D) Gene expression analysis of PDE and effector cytokine genes in T cell populations. (A) Naïve T cells from LN of TCR Tg donor mice (5C.C7/RAG-2^−/−^/CD45.1 B10.A) were injected i.p. into normal syngeneic CD45.2 B10.A recipient mice and activated by antigen using miniosmotic pumps. Memory cells were generated *in vivo* by antigen priming and boost using miniosmotic pumps as described [32]. The LN and SP were removed 20–22 h or 38–44 h later and single cell suspensions (i) were stained with (ii) PE Cy7 anti-CD4, (ii) PE anti TCR Vβ3, (iii) APC anti-CD45.2 and (iii) FITC anti-CD45.1. The Tg T cells were purified by FACS sorting of the CD4^+^/TCR Vβ3^+^/CD45.1^+^/CD45.2^−^-population as demonstrated for a representative sample (i–iii). (B) cDNAs were made from the sorted naïve and memory T cell populations and relative gene expression of (i) PDE3B, (ii) PDE4B, and (iii) PDE8A was analyzed by qRT-PCR. (C) CD4^+^CD25^−^ T cells were isolated and stimulated by plate-bound anti-CD3 mAb (5 µg/ml) *in vitro* for 18 h, and (D) splenocytes were activated for 48 h with Con A (3 µg/ml). Relative expression of PDE and effector cytokine genes was determined by qRT-PCR. Values are presented as mean + SEM of the ratio between target gene expression and RPL19 expression. Data are representative of two to three independent experiments performed in triplicate.

We have previously reported on PDE8 expression in unactivated and polyclonally stimulated splenocytes, but to date, no *in vivo* observations on PDE8 expression in T cells have been published [25]. To test this, we transferred CD4^+^ TCR transgenic (Tg) T cells into wildtype non-transgenic mice, activated naïve or memory Tg T cells with antigen *in vivo* [32], isolated Tg T cells (Fig. 1A) and analyzed their expression of PDE genes (Fig. 1B, Table S1). As expected, activated Tg T cells predominantly expressed PDE3 and PDE4 genes *in vivo* (Fig. 1Bi and ii). Our *in vivo* findings (Fig. 1B) are consistent with *in vitro* findings in isolated CD4^+^CD25^−^ Teff cells stimulated with anti-CD3 mAb (Fig. 1Ci) or T cell blasts derived from splenocytes activated with the mitogen Concanavalin A (Con A) (Fig. 1Di). In addition, CD4^+^ Tg T cells expressed PDE8A *in vivo* (Fig. 1Biii), in agreement with results from gene array analyses (S.Z.B.-S., unpublished data). Expression of the PDE8A gene, a PDE isoform with a very high affinity for cAMP (*K*m≈0.04–0.15 µM), in CD4^+^ T cells and T cell blasts activated *in vivo* and *in vitro* ranged between 20% and 50% of PDE3B and PDE4B expression levels (Fig. 1B, Ci and Di). Both anti-CD3 mAb activated CD4^+^CD25^−^ Teff cells and Con A activated T cell blasts expressed IFN-γ, TNF-α, and IL-2 genes (Fig. 1Cii and Dii). Overall, PDE and Th1 cytokine profiles between activated CD4^+^ T cells and T cell blasts were comparable and included the expression of PDE8A.

### Targeting PDE8 is required for rapid suppression of Teff cell adhesion to endothelial cells

**Figure 2:**
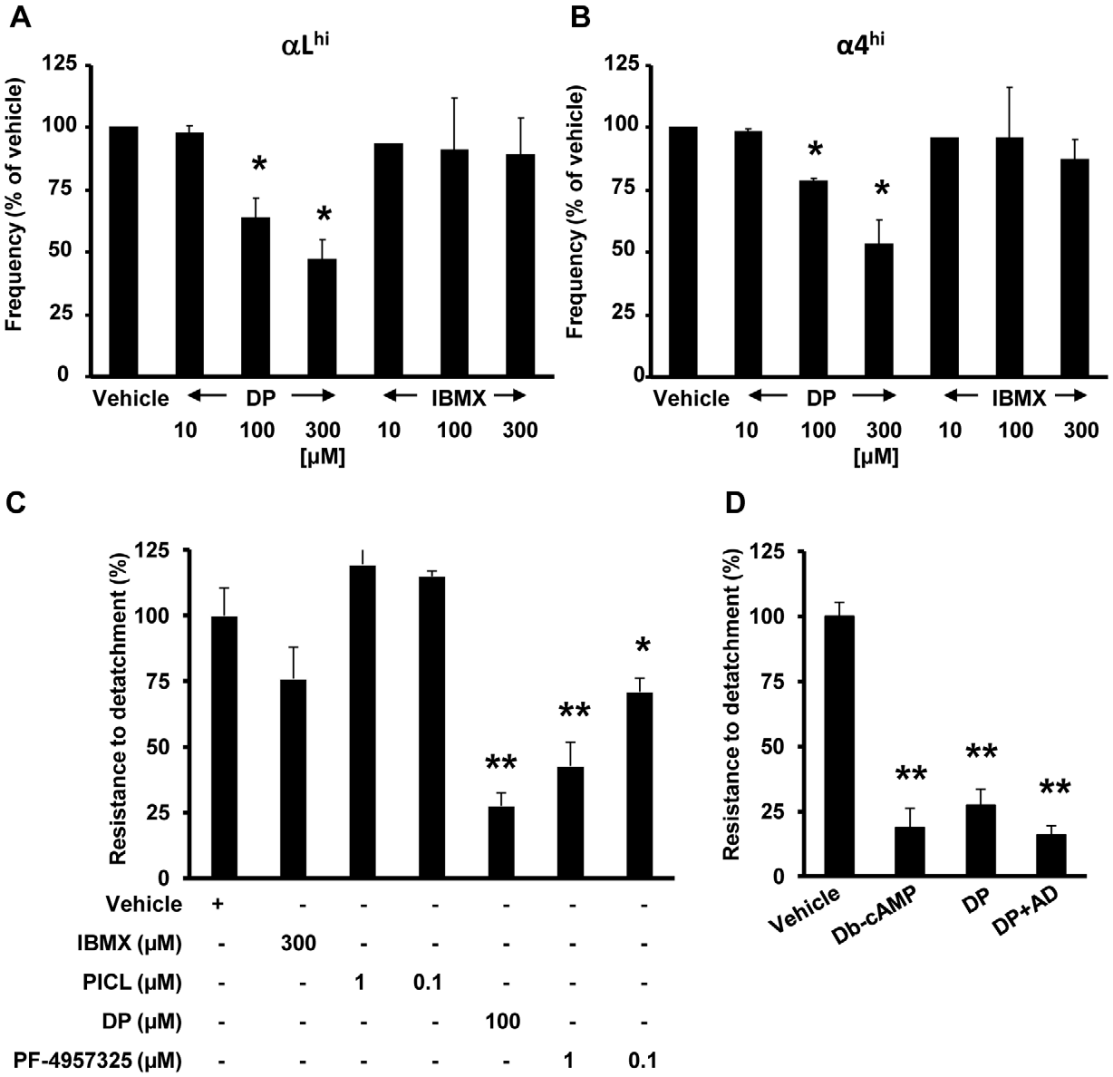
Inhibiting PDE8 suppresses integrin surface expression and adhesion of Teff cells to endothelial cells. (A and B) Modulation of αL and α4 integrin surface expression on Teff cells in response to PDE inhibitors. In both panels, CD4^+^CD25^−^ Teff cells were treated with DP or IBMX before analysis of CD4, αL, and α4 integrin cell surface expression by FACS. Cells were gated on the CD4^+^ population. Frequency of αL^hi^ (A) and α4^hi^ (B) Teff cells in response to different doses of PDE inhibitors normalized to the vehicle condition set at 100 percent. DP significantly reduced integrin surface expression while IBMX did not. (C and D) Inhibition of T cell blast adhesion to endothelial cells by DP and PF-4957325-00 within 90 min. T cell blasts from C57BL/6 mice and bEnd.3 endothelial cells were incubated with (C) IBMX (300 µM), PICL (1 or 0.1 µM), DP (100 µM) or PF-4957325-00 (1 or 0.1 µM), (D) dibutyryl-cAMP (Db-cAMP; 500 µM) or DP in the presence or absence of AD (1 U/ml) or vehicle (0.1% DMSO). Values are normalized to the vehicle condition and presented as the mean + SEM percentage of T cell blasts resistant to detachment. Data are representative of one to five independent experiments performed in triplicate (*p<0.05, **p<0.001; one-way ANOVA and Bonferroni t-test).

In activated T cells, heterodimeric integrin molecules containing the αL or α4 chain mediate critical interactions with endothelial cells [26], [27]. Regulation of integrin expression and function at the surface of lymphocytes and granulocytes by intracellular cAMP has previously been reported [28], [33], [34] Among a wide variety of PDE inhibitors tested against PDE8A, only dipyridamole (DP) was found to inhibit this enzyme with reported IC_50_s in the range of 4–9 µM [1], [11], [35], [36]. By exploring the selective ability of DP to modulate surface expression of the αL subunit of LFA-1 and α4 subunit of VLA-4 and α4β7 (Fig. S1) we attempted to define the role of PDE8 in regulating integrins involved in T cell extravasation and inflammatory diseases [26], [37], [38]. Following a 45-min incubation with 100 µM or 300 µM DP, the frequency of αL^hi^ and α4^hi^ Teff cells was significantly reduced as compared to the vehicle control or exposure to 10 µM DP (Fig. 2A and B). In contrast, IBMX, a non-specific PDE inhibitor which inhibits all known PDE gene families capable of hydrolyzing cAMP with the exception of PDE8 [1], [11], [35], [36] did not significantly reduce integrin surface expression (Fig. 2A and B). Following the establishment of dose-response curves (Fig. 2A and B) and based on others' and our previous studies [25], [39]–[42], we chose to use DP at a concentration of 100 µM and IBMX at a concentration of 300 µM in all further assays. To define the role of PDE isoforms in the regulation of T cell interaction with endothelium, we next tested PDE inhibitors in T cell blast-endothelial cell adhesion assays (Fig. 2C and D). DP rapidly reduced adhesion of T cell blasts to bEnd.3 endothelial cells by 73% (Fig. 2C and D and Fig. S4) (*p<0.05, **p<0.001; one-way ANOVA and Bonferroni *t*-test). In accordance with results from FACS analysis of integrin surface expression, an inhibitory effect was observed with DP while IBMX did not significantly reduce adhesion (Fig. 2C). In addition, the potent and highly PDE4-selective inhibitor piclamilast (PICL; IC_50_ = 0.001 µM) (Fig. 2C) and the PDE3-selective inhibitor motapizone (A.G.V. and S.B., unpublished data) also failed to suppress adhesion in our assays. Our results are in agreement with a previous report demonstrating no significant effect of rolipram on the adhesion of activated T cells to immobilized VCAM-1 and endothelial cells for up to 8 h of exposure [43]. To further probe the selectivity of PDE8 action in T cell adhesion, we evaluated our findings with the recently developed PDE8-selective inhibitor PF-4957325-00 (IC_50_ = 0.0007 µM for PDE8A and <0.0003 µM for PDE8B; Table 1, Fig. S2). As with DP, the PDE8-selective inhibitor PF-4957325-00 suppressed T cell blast adhesion to endothelial cells by 57 and 29% at 1 µM and 0.1 µM, respectively (Fig. 2C) (*p<0.05, **p<0.001; one-way ANOVA and Bonferroni *t*-test). These results on adhesion are notable since in proliferation studies, PICL was significantly more efficient at suppressing Teff cell proliferation compared to PF-4957325-00, i.e. 95% vs. 43% at 1 µM, respectively (Fig. S3). Our data suggest a selective effect of PDE8 inhibition on rapid Teff cell adhesion to endothelial cells.

**Table 1:** *In vitro* potency of the PDE8-selective inhibitor PF-4957325-00.

PDE	IC_50_
8A	0.0007 µM
8B	<0.0003 µM
All other PDE isoforms	>1.5 µM

We next assessed whether the DP effect is consistent with signaling through the cAMP pathway, and thus its action as a PDE inhibitor. PKA inhibits integrin surface expression and avidity on leukocytes and spatially controls α4 integrin phosphorylation required for efficient cell migration [44]–[46]. In our experiments, dibutyryl-cAMP (Db-cAMP), an agonistic cAMP analog, reduced T cell blast adhesion to endothelial cells by 81% (Fig. 2D), comparable to the 73% reduction resulting from DP treatment (*p<0.05, **p<0.001; one-way ANOVA and Bonferroni *t*-test).

Besides inhibiting PDE activity, DP blocks the reuptake of extracellular adenosine which can also increase cAMP synthesis in T cells. To determine if this mechanism accounts for some of the action of DP, we tested the effect of adenosine deaminase (AD) (1 U/ml) which has been shown to degrade extracellular adenosine at this concentration, in the adhesion assay. AD did not reverse the inhibitory effect of DP on T cell blast adhesion. Treatment with DP in the presence of AD reduced adhesion by 84% (*p<0.05, **p<0.001; one-way ANOVA and Bonferroni *t*-test), similar in magnitude to inhibition obtained with DP alone (Fig. 2D). These results indicate that DP is not acting through an effect on extracellular adenosine and support our conclusion that DP acts as a PDE inhibitor in our experiments.

The recruitment chemokine CXCL12 has been widely shown to promote adhesion strengthening between T cell integrins and their vascular ligands after CXCL12 immobilization on the luminal surface of endothelial cells [26], [27]. To test whether CXCL12 could overcome the inhibitory effect of DP on T cell blast-endothelial cell adhesion, we preincubated activated bEnd.3 cells with CXCL12 (250 ng/ml) and DP (100 µM) for 45 min before performing the adhesion assay. Our results show that CXCL12 did not reverse the inhibitory effect of cAMP signaling activated by DP (Fig. S4).

### DP treatment causes a short increase of intracellular cAMP followed by a compensatory increase of PDE4B gene expression and suppression of Th1 cytokines in Teff cells

**Figure 3:**
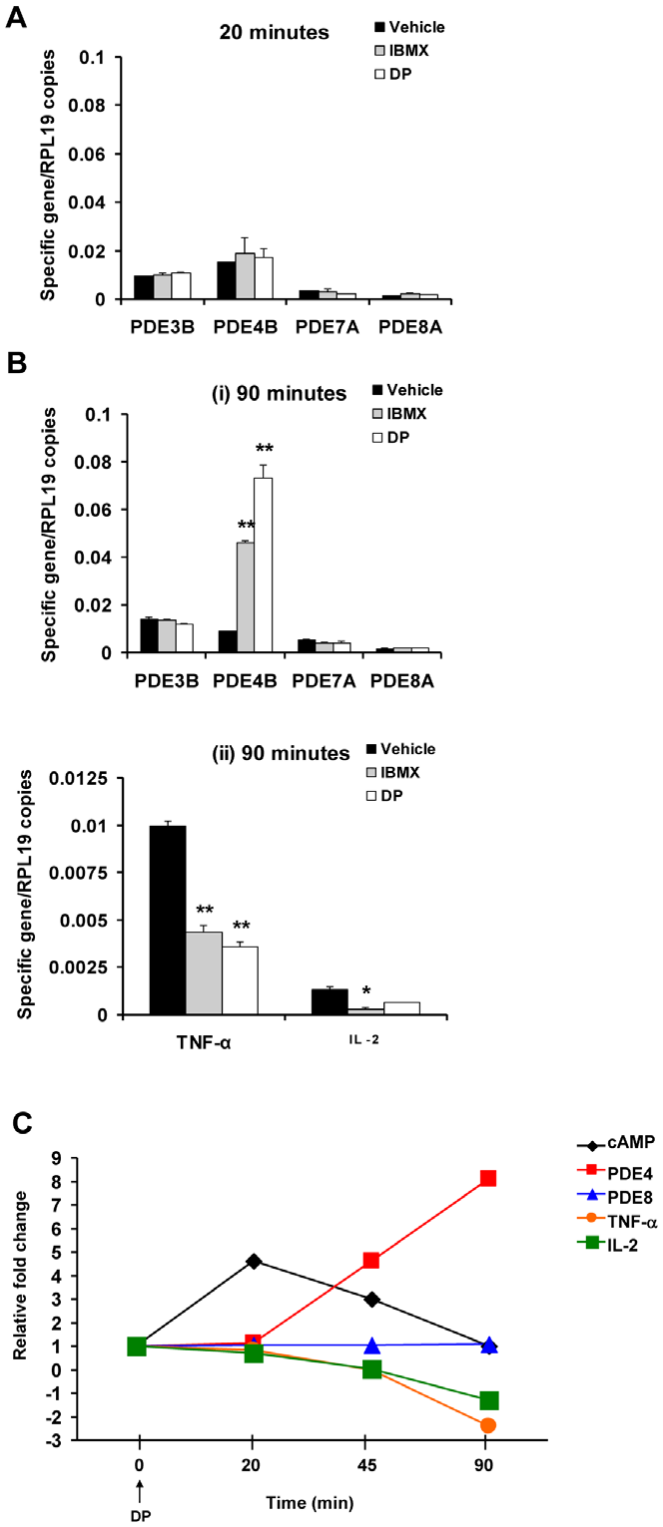
DP treatment inhibits TNF-α and IL-2 gene expression and causes a compensatory increase in PDE4B expression. (A and B) The expression profile of PDE and cytokine genes in Teff cells following treatment with PDE inhibitors. Purified CD4^+^CD25^−^ T Teff cells were incubated with IBMX (300 µM), DP (100 µM) or vehicle (0.1% DMSO) for (A) 20 or (B) 90 min. PDE (A, Bi) and cytokine (Bii) relative gene expression was analyzed by qRT-PCR. Values are presented as mean + SEM of the ratio between target gene expression and RPL19 expression. Data are representative of two independent experiments performed in triplicate (*p<0.05, **p<0.001; one-way ANOVA and Bonferroni *t*-test). (C) Summary of the kinetics of intracellular cAMP levels, PDE and cytokine gene expression after exposure of Teff cells to DP (100 µM). Values are presented as relative fold-change compared to vehicle control at 0 min (set as 1) and data are representative of one to three independent experiments.

PDEs are dynamic regulators and rapidly respond to changes in cAMP levels [1], [2]. We found that PDE4B expression was selectively increased after DP and IBMX treatment, whereas PDE3B, 7A, and 8A expression were unchanged. Initially unchanged at 20 min (Fig. 3A), PDE4B gene expression increased 8-fold in CD4^+^CD25^−^ Teff cells after 90 min of DP treatment and 5-fold after IBMX treatment (Fig. 3Bi) (*p<0.05, **p<0.001; one-way ANOVA and Bonferroni *t*-test). Of note, we observed an increase of cAMP at 20 min of DP treatment which was resolved by 90 min (Fig. 3C). Theses data are the first demonstration of a compensatory upregulation of PDE4B gene expression in response to DP action in Teff cells. Despite the increase in PDE4B gene expression, DP decreased gene expression of TNF-α by 3-fold and IL-2 by 2-fold in Teff cells after 90 min of exposure. Similar results were obtained with IBMX (Fig. 3Bii) (*p<0.05, **p<0.001; one-way ANOVA and Bonferroni *t*-test). Thus, DP action on Teff cells causes an increase in cAMP levels after 20 min, and subsequently a change in expression of PDE4 and Th1 cytokine genes after 90 min (Fig. 3C).

### DP suppresses proliferation of Teff cells in the absence of ICER

**Figure 4:**
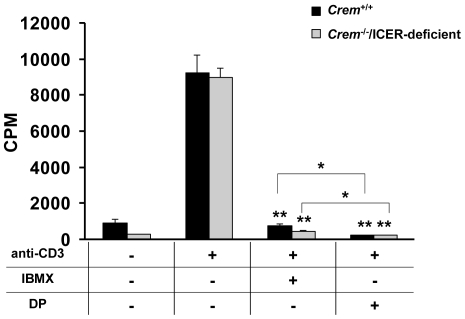
DP inhibits proliferation of *Crem* ^−/−^/ICER-deficient Teff cells. Proliferation of purified *Crem* ^+/+^ or *Crem* **^−/−^** derived CD4^+^CD25^−^ Teff cells exposed to PDE inhibitors. Teff cells (5×10^4^/well) were cocultured with irradiated T cell-depleted splenocytes presenting soluble anti-CD3 mAb or control in the presence of IBMX (300 µM), DP (100 µM), or vehicle control. The extent of proliferation was determined by [^3^H]thymidine incorporation at 64 h and results are presented as mean + SEM counts per min (cpm). Data are representative of two independent experiments performed in triplicate (*p<0.05, **p<0.001; comparisons to vehicle were analyzed using a one-way ANOVA and Bonferroni *t*-test; comparisons between DP and IBMX were performed using an unpaired *t*-test).

Since PDE4-selective inhibitors show limited effects on T cell proliferation [9] we next tested DP in proliferation assays of CD4^+^CD25^−^ Teff cells. In our assays, both IBMX and DP potently suppressed Teff cell proliferation, but the inhibitory action of DP was greater (Fig. 4) (*p<0.05, **p<0.001; one-way ANOVA and Bonferroni *t*-test). Multiple mechanisms have been suggested for the suppression of T cell function by cAMP, including induction of the transcription factor ICER [47]. ICER is transcribed from an alternative cAMP-inducible promoter of the *Crem* gene. Based on the results of our experiments, we directly addressed the role of ICER in DP and IBMX mediated Teff cell suppression by using *Crem* ^−/−^ mice which lack ICER [48]. We found that gene deletion of *Crem* in Teff cells (*Crem* ^−/−^/ICER-deficient Teff cells) did not affect DP mediated suppression of proliferation (Fig. 4) or Th1 cytokine gene expression (A.G.V. and S.B., unpublished data). The viability of Teff cells was not affected by the DP treatment (A.G.V. and S.B., unpublished data). Taken together, our results suggest a role for PDE8 in controlling Teff cell proliferation and indicate that the transcriptional repressor ICER is not required for cAMP mediated suppression.

### Endothelial cells express PDE8A

**Figure 5:**
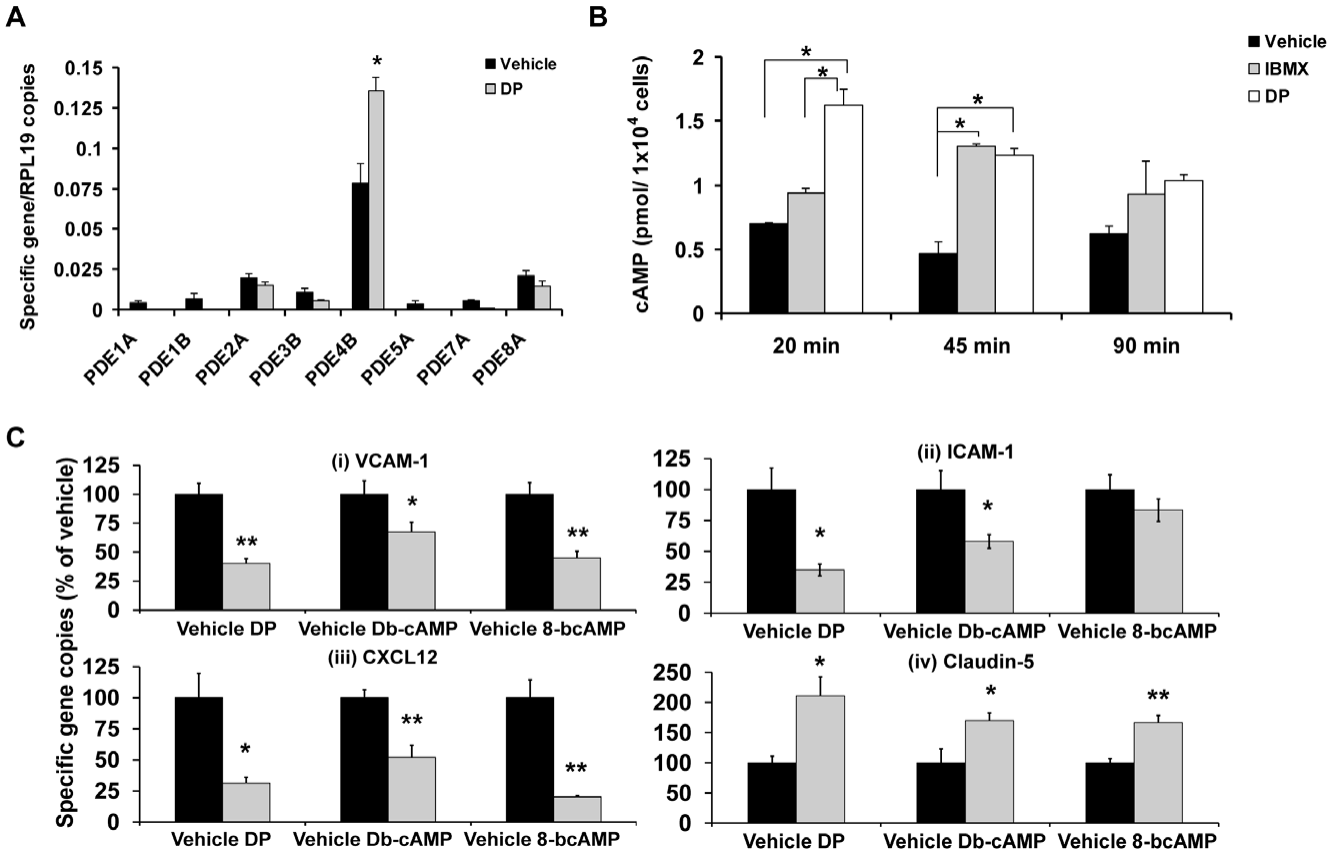
DP rapidly induces cAMP and modulates expression of genes involved in the regulation of vascular recruitment and barrier functions in endothelial cells. (A) Relative PDE gene expression in activated bEnd.3 endothelial cells incubated with DP (100 µM) or vehicle (0.1% DMSO) for 45 min. Values are presented as mean + SEM of the ratio between target gene expression and RPL19 expression. Data are representative of three independent experiments performed in triplicate (*p<0.05, **p<0.001; unpaired *t*-test). (B) cAMP content of activated bEnd.3 cells incubated with IBMX (300 µM), DP (100 µM), or vehicle (0.1% DMSO) for 20, 45, and 90 min. Values are presented as mean + SEM pmol cAMP per 10^4^ cells determined by ELISA. Data are representative of two independent experiments performed in duplicate (*p<0.05, **p<0.001; one way ANOVA and Bonferroni *t*-test for comparisons between vehicle and DP or IBMX and unpaired *t*-test for comparisons between DP and IBMX). (C) Modulation of (Ci) VCAM-1, (Cii) ICAM-1, (Ciii) CXCL12, and (Civ) claudin-5 gene expression in activated bEnd.3 cells by DP (100 µM), dibutyryl-cAMP (Db-cAMP; 500 µM), 8-bromo-cAMP (500 µM), or vehicle (0.1% DMSO, or media) for 45 min. Values are shown as mean + SEM relative target gene expression in treated sample as percentage of vehicle control. Data are representative of three independent experiments performed in triplicate (*p<0.05, **p<0.001; unpaired *t*-test).

To more fully elucidate the role of PDE8 in rapid cAMP signaling during T cell–endothelial cell interaction, we extended our investigations to analyze PDE expression in endothelial cells. We confirmed expression of PDE1, 2, 3, 4, 5 and 7 genes in bEnd.3 cells [49], [50]. Importantly, we discovered considerable expression of PDE8A in these cells (Fig. 5A). Similar to Teff cells, PDE4B was the most abundantly expressed PDE gene in bEnd.3 cells. In contrast, PDE8A expression was 4-fold lower (Fig. 5A). Nevertheless, the expression level of PDE8 was comparable to that of PDE2A which was shown to be functionally important in vascular beds despite its lower abundance [30]. As in Teff cells (Fig. 3B), activation of cAMP signaling through DP treatment in bEnd.3 cells induced a compensatory increase of PDE4B expression while expression of other PDE genes, including PDE8A, was not significantly altered (Fig. 5A) (*p<0.05, **p<0.001; unpaired *t*-test).

### DP rapidly increases cAMP levels in endothelial cells

Raising cAMP levels through PDE inhibition in endothelial cells has been shown to increase barrier function and down regulate expression of adhesion molecules [29]–[31]. Here, we tested the ability of DP and IBMX to increase cAMP levels in bEnd.3 cells. DP, but not IBMX, increased cAMP levels by over 2-fold within 20 min (Fig. 5B). At 45 min cAMP levels were significantly increased by both DP and IBMX (Fig. 5B) (*p<0.05, **p<0.001; one-way ANOVA and Bonferroni *t*-test). Hence, cAMP is increased more rapidly by DP than IBMX.

### DP suppresses gene expression of vascular T cell recruitment molecules and induces the tight junction molecule claudin-5

To further explore the response of endothelial cells to DP, we tested whether a DP-mediated increase in cAMP caused changes in gene expression of molecules involved in vascular recruitment of T cells and the formation of endothelial tight junctions. DP reduced gene expression of VCAM-1 (Fig. 5Ci), a vascular adhesion molecule promoting integrin-dependent adhesive interactions of T cells with venules, by 60%. The reduction of VCAM-1 expression by DP is consistent with the action of two cell permeable analogs of cAMP, dibutyryl-cAMP and 8-bromo-cAMP which suppressed VCAM-1 expression by 33% and 55%, respectively (Fig. 5Ci). DP also reduced gene expression of the vascular adhesion molecule ICAM-1 by 65%, again consistent with cAMP-dependent effects as dibutyryl-cAMP inhibited ICAM-1 by 42% (Fig. 5Cii). Since CXCL12 is a strong recruitment chemokine for T cells [27], we investigated the effect of DP on its expression in endothelial cells. DP reduced CXCL12 by 69% while dibutyryl-cAMP and 8-bromo-cAMP reduced CXCL12 by 48% and 80%, respectively (Fig. 5Ciii). In addition to its suppression of vascular adhesion molecules and chemokines, the anti-inflammatory action of cAMP is associated with an increase in endothelial barrier integrity [29]–[31]. Therefore, we examined whether DP caused upregulation of claudin-5 expression, a critical component of endothelial tight junctions whose activity is required for endothelial barrier function [51]. Our results show that DP increased claudin-5 gene expression by 111%, while dibutyryl-cAMP and 8-bromo-cAMP increased claudin-5 expression by 70% and 67%, respectively (Fig. 5Civ). Thus, inhibition of PDEs by DP suppressed expression of molecules promoting adhesive interactions between leukocytes and vascular endothelium while increasing the expression of claudin-5, an adhesion molecule critical to the formation of tight endothelial junctions (*p<0.05, **p<0.001; unpaired *t*-test).

### DP treatment reduces endothelial cell CXCL12 gene expression at the microvasculature *in vivo*

**Figure 6:**
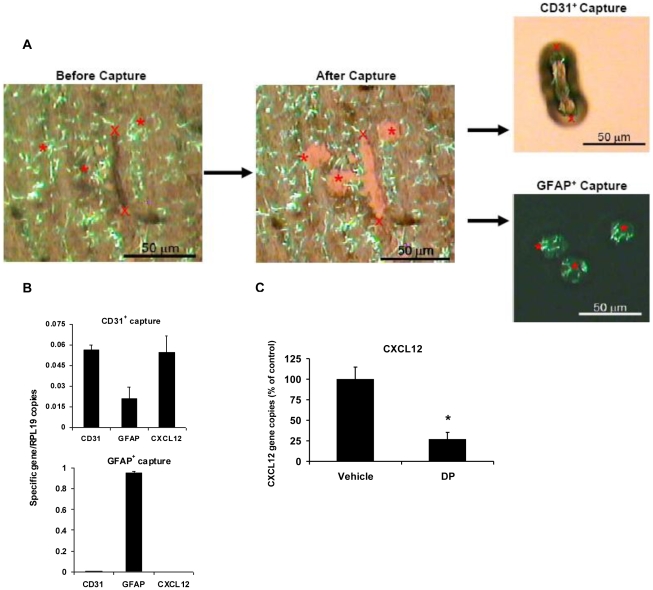
Treatment with DP *in vivo* inhibits gene expression of CXCL12 in microvascular endothelial cells. (A) Isolation of endothelial cells and astrocytes *in situ* by laser-capture microdissection. An immunostained cerebellar cryosection from which CD31 positive microvascular endothelial cells (anti-CD31 mAb/peroxidase-DAB) and GFAP positive astrocytes (anti-GFAP mAb/Alexa Fluor 594) were selectively captured is shown. (B) CXCL12 gene expression in endothelial cells from mice treated *in vivo* with DP. After selected tissue areas were captured, gene expression of CD31, GFAP, and CXCL12 in CD31 positive endothelial cell captures (top panel) and in GFAP positive astrocytic captures (bottom panel) was analyzed by qRT-PCR. (C) The effect of *in vivo* treatment with DP on gene expression of CXCL12 in endothelial cells. C57BL/6 mice were given two i.p. injections of DP (1 mg) or vehicle (0.1% DMSO). CXCL12 gene expression in CD31 positive endothelial cell captures from cerebellar cryosections 30 min after the last DP treatment is shown. Values are presented as mean + SEM relative CXCL12 gene expression in cell captures from DP treated mice as percentage of the vehicle control treatment group. Data represent 9000 individual cell-selective captures from at least 20 immunostained cerebellar cryosections prepared from 2 animals per treatment group (*p<0.05, **p<0.001; unpaired *t*-test). Microphotographs were taken on an Olympus IX51 inverted microscope at an original magnification of 200x.

CXCL12 is an efficient vascular recruitment chemokine. Following our *in vitro* demonstration that CXCL12 mRNA in endothelial cells was reduced by short term exposure to DP, we tested the effect of DP on microvascular endothelium *in vivo* by use of laser-capture microdissection (LCM). To facilitate selective capture of endothelial cells by LCM, we employed a staining procedure that enabled us to spatially resolve endothelial cells, i.e. CD31^+^ cells, from the perivascular border of the glia limitans, i.e. GFAP^+^ astrocytic end feet [52]. To ensure our dissected samples were highly enriched with microvascular endothelial cells, we initially evaluated LCM cDNA by analyzing the ratio of CD31/GFAP copies. A representative analysis is shown (Fig. 6A and B) for microvessel-derived (CD31^+^) and for astrocytic captures, with the microvessel cDNA containing 2.5-times as many CD31 transcripts as GFAP gene copies. Conversely, astrocyte-selective (GFAP^+^) captures expressed 317-times as many GFAP transcripts as CD31 gene copies. Furthermore, astrocytes contained no detectable CXCL12 mRNA, while microvessels readily expressed CXCL12 (Fig. 6B). The lack of significant CXCL12 transcripts in resting astrocytes is further evidence that the CXCL12 mRNA was derived from dissected microvessels. Using this approach, we were able to measure the effect of DP injection on gene expression in vascular endothelial cells of the blood-brain barrier *in vivo*. Our results show that DP treatment reduced CXCL12 gene expression (73%) in mouse brain microvessels (Fig. 6C) (*p<0.05, **p<0.001; unpaired *t*-test).

## Discussion

Our results indicate a non-redundant role for PDE8 during regulation of T cell adhesion to vascular endothelium through the cAMP signaling pathway. Our analysis demonstrates for the first time that activated CD4^+^ T cells express PDE8 *in vivo*. The data further suggest that targeting PDE8 through the use of the PDE inhibitor DP is critical to rapidly control adhesion and directed migration of activated T cells. Remarkably, despite abundant expression of PDE3 and PDE4 in T cells, selective inhibition of these PDE isoforms fails to inhibit rapid T cell adhesion. In addition to its immediate effects on T cell adhesion, DP suppresses CD4^+^CD25^−^ Teff cell proliferation and Th1 cytokine production. Besides targeting T cells, DP acts on endothelial cells by altering gene expression of adhesion, chemotactic and tight junction molecules *in vitro* and *in vivo*. This two pronged control of T cell-endothelial cell interaction by DP indicates that PDE8 may serve as a novel target to suppress recruitment of activated T cells from the bloodstream into tissues during an inflammatory response.

cAMP is the prototypical second messenger which impacts on almost every aspect of cell activity and exerts myriad yet specific effects on cell functions [53]. The ability to form site- and function-specific cAMP gradients within the cell critically depends on its degradation by PDEs which are pivotal regulators of intracellular cAMP activity. Observations that inhibition of PDE4, the most abundantly expressed PDE in T cells, blocks T cell activation and function through elevating cAMP, prompted the development of PDE4 inhibitors as potential immunosuppressive therapies [10], [11], [18], [23], [24]. However, none has yet been approved for clinical use [11], [18], [23], [24] and rolipram failed to suppress CNS inflammation in MS patients as measured by brain MRI [21]. The recent discovery of novel PDE variants in T cells [4]–[6] suggested that individual PDE isoforms may serve to modulate distinct regulatory pathways [25]. These findings led us to hypothesize that PDE4-selective inhibitors may have shown limited efficacy because important PDE isoforms in activated T cells were not targeted.

To identify potential PDE targets in T cells other than PDE4, we first analyzed expression of PDE isoforms *in vivo*. Based on initial detection of PDE8 expression in a gene array screen (Z.S.B.-S., unpublished data), we determined PDE8 expression by qRT-PCR in Teff cell populations and CD4^+^ T cells activated by specific antigen *in vivo*. We found that *in vivo*-activated naïve and memory T cells and *in vitro*-activated Teff cells express PDE8A at lower levels than PDE3B and PDE4B. Despite its lower expression, the high affinity of PDE8A for cAMP and effects of intracellular compartmentalization could account for its critical role in regulating T cell functions [13], [35], [36]. Thus, our findings that PDE8A expression levels are comparable between T cells activated by specific antigen *in vivo* and polyclonally activated T cells *in vitro* suggest a role for the PDE8 family in regulating cAMP signaling in these cells.

We next asked whether PDE8 controls integrin expression on CD4^+^CD25^−^ Teff cells and thus may play a non-redundant role in T cell adhesion to vasculature – both functions which are known to be regulated by cAMP [29]. Among molecular pathways that regulate T cell extravasation, cAMP is of particular interest as it is generated in both leukocytes and endothelial cells and regulates leukocyte chemotaxis as well as endothelial barrier function in blood and lymphatic vessels [29]–[31], [54]. Previously, we found that the broad, non-selective PDE inhibitor IBMX produced little inhibition of directed migration of activated T cells towards the chemokine CXCL12 [25]. Only the PDE inhibitor DP [35], [36], [55] strongly inhibited migration of activated T cells. The spectrum of PDEs targeted by DP includes PDEs 4-8, 10 and 11 [1], [11], [56], thus including the critical PDE8 isoforms. It is important to note that T cell motility and firm attachment to vascular ligands are differentially regulated. Unlike migration within tissue parenchyma [26], T cell attachment to endothelial cells is exposed to disruptive shear forces and is essentially controlled by the function of LFA-1 (αLβ2) and the α4 integrins VLA-4 and α4β7. We found that expression of integrins controlling shear-resistant vascular attachment of T cell blasts is significantly reduced by DP treatment compared to IBMX treatment (Fig. 2A and B).

With regard to integrin functions, recent studies demonstrated that adhesion of T cells can be blocked by extended treatment (8–48 h) with the PDE4-selective inhibitor rolipram [43]. To our surprise, we failed to detect any suppressive effect of the highly potent PDE4-selective inhibitor PICL on T cell adhesion to activated endothelial cells within 90 min. In contrast, DP reduced adhesion of T cell blasts by 73% while PF-4957325-00 reduced adhesion by a maximum of 53% (Fig. 2C and D). A possible explanation for the differences in these observations is that PDE4-selective inhibitors require long term exposure of T cells to achieve an inhibitory effect on T cell adhesion since exposure of T cells to rolipram for a period of less than 8 h had no effect on their adhesion to vascular ligands or endothelial cells [43]. Thus, results from previous studies are consistent with our observations. Even under the conditions of long-term exposure, it is notable that DP inhibited proliferation of CD4^+^CD25^−^ Teff cells more potently than IBMX, and that these immunosuppressive effects were independent of the cAMP induced transcriptional repressor ICER (Fig. 4). However, PICL was also very efficient at suppressing proliferation (Fig. S3), while PF-4957325-00 was less potent than PICL in this assay. Thus, our data suggest that a rapid effect on T cell adhesion critically depends on a PDE inhibitor that blocks PDE8 enzymatic activity, while inhibition of Teff cell proliferation is less dependent on blocking the PDE8 isoform. At present, it is unknown what accounts for the different short-term versus long-term effects of selected PDE isoform inhibition during adhesion and proliferation. A possible mechanism may be that DP and PF-4957325-00 upregulate intracellular cAMP levels more rapidly and efficiently than PDE-selective inhibitors that do not block PDE8, requiring a longer time of action for less efficient PDE inhibitors during Teff cell adhesion [57]. Since PDE8A is a very high affinity cAMP-specific PDE with a *K*m value ranging from 0.04–0.15 µM, 40–100 times lower than that of PDE4, it is likely to be functioning at lower cAMP concentrations than PDE4 and may thus be involved in the control of intracellular cAMP concentrations at basal levels and in the immediate response to acute increases of cAMP in specific cell regions [35], [36], [58]. This mechanism would be consistent with our data.

DP has other actions in addition to its inhibition of selected PDEs [42], including suppression of adenosine uptake into cells [59], thereby potentially increasing extracellular adenosine available in culture medium. To exclude the action of extracellular adenosine in our assay systems, we tested the effect of DP in the presence of AD which inactivates adenosine. Both in chemotaxis [25] and adhesion assays (Fig. 2D), extracellular adenosine was not responsible for the inhibitory effect of DP, suggesting DP is indeed acting through PDE inhibition.

In endothelial cells, PDEs are critical in regulating barrier permeability [29]–[31]. In agreement with previous reports [49], [50], we find expression of PDE2, PDE3, and abundant expression of PDE4 in bEnd.3 cells. In addition, we report for the first time PDE8A expression in mouse endothelial cells. We demonstrate that inhibiting PDEs with DP decreased gene expression of VCAM-1 and ICAM-1, as well as CXCL12 in endothelial cells. In striking contrast to the downregulation of vascular adhesion molecules VCAM-1 and ICAM-1 that mediate T cell integrin interactions, DP increased gene expression of claudin-5 (Fig. 5C), an intercellular adhesion molecule that is a marker for endothelial tight junctions [51], [60]. Its function is non-redundant as claudin-5 is the major claudin identified in normal endothelial cells. Of note, *claudin-5^−/−^* mice have a defective blood-brain barrier [60]. We demonstrate here that DP upregulates cAMP in endothelial cells, and that cAMP analogs mimic DP effects on endothelial gene expression. Taken together, DP exerts a two way control of endothelial function under inflammatory conditions by inhibiting expression of T cell recruitment molecules and increasing expression of the tight junction molecule claudin-5.

As isolated microvessels and endothelial cells undergo significant changes in culture compared to their features *in vivo* [61], we tested the effect of DP on the brain microvasculature *in situ* using LCM. Confirming our *in vitro* observations, administering DP *in vivo* significantly reduced CXCL12 gene expression (Fig. 6C). This result demonstrates the feasibility of cell-selective LCM coupled to gene expression analysis to measure drug effects on the blood-brain barrier, and specifically supports the concept that the PDE inhibitor DP has anti-inflammatory action in this vascular bed [42]. Together with our data on PDE expression analysis of T cells *in vivo*, these results suggest that PDE8 is an important target for inhibiting the recruitment of activated T cells to vascular endothelium by regulating cAMP signaling in both cell types.

To date, no PDE8-selective inhibitors had been available, and thus far, DP has been the most potent agent reported to inhibit it [35], [36]. In addition to testing DP, our study now presents for the first time the characterization of a novel and previously untested PDE8-selective inhibitor, PF-4957325-00, in Teff cell adhesion and proliferation studies. It is noteworthy that blocking the α4 subunit of VLA-4 on Teff cells by antibody provides a highly efficient way to treat numerous autoimmune diseases in animal models and in humans, including multiple sclerosis [62]–[67]. Based on our study, efforts to develop and test selective inhibitors of PDE8 such as PF-4957325-00 should be undertaken as a means to develop novel therapeutic agents for treatment of inflammatory disorders associated with the vascular recruitment of activated T cells [68]–[72].

## Materials and Methods

### Ethics Statement

All mouse experiments were performed according to IACUC-approved and -supervised protocols at UCHC and NIH.

### Animals

6–12 wk old female C57BL/6 mice were from Jackson Laboratories, Bar Harbor, ME. *Crem* ^−/−^mice were bred as previously described [48]. 5C.C7/RAG-2^−/−^/CD45.1 B10.A and CD45.2 B10.A mice were from Taconic Farms Inc. (Hudson, NY) [32].

### Cell culture

Con A activated mouse splenocytes as a source of T cell blasts were prepared and cultured as described [25]. CD4^+^CD25^−^ Teff cells were separated from CD4^+^CD25^+^ Treg cells using a CD4^+^CD25^+^ Regulatory T Cell Isolation Kit (Miltenyi Biotec, Auburn, CA) and activated for 18 h on plate-bound anti-CD3 mAb (5 µg/ml). Cells of the murine brain endothelium-derived cell line bEnd.3 (ATCC, Manassas, VA) were seeded into 24-well plates (Costar, Cambridge, MA) in DMEM supplemented with 100 U/ml penicillin, 100 mg/ml streptomycin, 2 mM L-Glutamine, and 10% fetal bovine serum (all Gibco, Carlsbad, CA). All bEnd.3 cell assays were performed on confluent monolayers of cultured cells. Endothelial cell passage numbers did not exceed 25.

### Generation and isolation of activated CD4+ T cells *in vivo*

Naïve T cells (2×10^6^) from LN and SP of Tg donor mice (5C.C7/RAG-2-/-/CD45.1 B10.A) were injected i.p. into normal syngeneic CD45.2 B10.A recipients. The mice were immunized 7–10 days later by implantation of 3 day miniosmotic pumps (Durect, Cupertino, CA) containing 400 µg of antigen (pigeon cytochrome C [PCC], Sigma-Aldrich, Springfield, MO) in HBSS [32]. The LN and SP were removed 20–22 or 38–44 h later and the single cell suspensions were stained with FITC anti-CD45.1, PE anti-Vβ3, APC anti-CD45.2 and PE Cy7 anti-CD4 (BD Biosciences, San Jose, CA). The Tg T cells were purified by FACS sorting of the CD4^+^/Vβ3^+^/CD45.1^+^/CD45.2^−^ population and the purity of the viable sorted Tg T cells was >90%. Induction and characterization of memory T cells were performed as previously described [32]. Briefly, memory cells were generated *in vivo* by priming transferred Tg T cells through implantation of 7 day miniosmotic pumps containing 1 mg of antigen (PCC) in HBSS. The mice were boosted at least 3 months after priming by implantation of 3 day miniosmotic pumps containing 400 µg of antigen (PCC) in HBSS. The LN and SP were removed 20–22 or 38–44 h later and the single cell suspensions stained with FITC anti-CD45.1, PE anti-Vβ3, APC anti-CD45.2 and PE Cy7 anti-CD4. Memory T cells were identified by memory markers as described [32]. The Tg T cells were purified by FACS sorting of the CD4^+^/Vβ3^+^/CD45.1^+^/CD45.2^−^ population; purity of the viable sorted Tg T cells was >90%.

### α4 and αL integrin cell surface staining

CD4^+^CD25^−^ Teff cells were treated with reagents as described in Cell treatment and then stained with the following antibodies: FITC α-CD4 (GK1.5), Biotin α-CD49d (R1-2) followed by Streptavidin-APC to detect α4 integrin, and PE α-CD11a (2D7) to detect αL integrin, or IgG2a,κ and IgG2b,κ as isotype controls (all Biolegend, San Diego, CA). FACS analysis was performed on a FACSCalibur 1 instrument (Becton Dickinson, San Jose, CA). Teff cells expressing high levels of αL or α4 integrin on their surface (αL^hi^ or α4^hi^ cells) were defined as the cell population staining with a mean florescence intensity above 10^2^ (gated on activated lymphocytes).

### RNA isolation and cDNA synthesis

Sorted Tg T cells from PCC stimulated or unimmunized mice were lysed in TRIzol (Invitrogen, Carlsbad, CA), RNA extracted with the RNeasy kit and genomic DNA removed using the RNase-Free DNase kit (Qiagen, Valencia, CA). RNA quality was evaluated by the Agilent 2100 Bioanalyzer. RNA from cells was isolated using the RNeasy mini kit. RNA from LCM studies was isolated using TRIzol and 4 µg glycogen (Ambion, Austin, TX) as a RNA carrier. RNA from cells and LCM captures were treated with Turbo DNA-free Dnase (Ambion). cDNA was synthesized using Superscript III reverse transcriptase (Invitrogen).

### Quantitative real-time RT-PCR analysis

10 ng cDNA, or 2 µl cDNA for LCM studies, was amplified by qRT-PCR in a 25 µl reaction using SYBR Green PCR Master Mix (Applied Biosystems, Foster City, CA). Primers were designed using Primer Express software v3.0 and primer efficiency verified by slope analysis to be 100%±2.5%. qRT-PCR was performed using an ABI 7500 fast system and data analyzed using the Δ^ct^ method (SDS software v3.0). Primer sequences (Invitrogen and IDT, Coralville, IA) are listed in Table S1. Amplicon sizes were approximately 100 bp.

### Cell treatment

In dose-response assays, Teff cells were exposed to 1–300 µM DP, 10–500 µM IBMX, and 0.01–3 µM PICL as indicated for 45 min. In assays utilizing endothelial cells, confluent bEnd.3 monolayers were activated with 200 ng/ml TNF-α (Peprotech, Rocky Hill, NJ) at 37°C for 2 h. For adhesion assays or qRT-PCR analysis, 100 µM DP in the presence or absence of 1 U/ml adenosine deaminase, 300 µM IBMX, 500 µM 8-bromo-cAMP (all Sigma-Aldrich), 500 µM dibutyryl-cAMP (Biomol, Plymouth Meeting, PA), 250 ng/ml CXCL12 (Peprotech), DMEM media or 0.1% DMSO in DMEM media as vehicle controls, 1 and 0.1 µM motapizone, PICL, or PF-4957325-00 were added to bEnd.3 cells for the last 45 min of TNF-α incubation. The PDE8-selective inhibitor PF-4957325-00 was synthesized by Pfizer Inc, Groton Laboratories, Groton, CT (Table 1, Fig. S2). PF-04957325-00 inhibits PDE8A with an IC_50_ = 0.0007 µM and PDE8B with an IC_50_ <0.0003 µM. The IC_50_ of PF-04957325 for members of all other PDE families is >1.5 µM. Additionally, PF-04957325 shows no activity in an off target selectivity panel screened at single dose at 10 µM concentrations. The PDE3- and PDE4-selective inhibitors motapizone and PICL were supplied by Drs. Christof Zitt and Armin Hatzelmann (Nycomed, Konstanz, Germany). T cell blasts or Teff cells were treated with the same reagents for 20, 45, or 90 min.

### *In vivo* DP treatment

0.4 ml of DP solution (1 mg DP in PBS/0.1% DMSO) or vehicle control (PBS/0.1% DMSO) were injected into C57BL/6 mice at 0 and 4 h. Mice were sacrificed by CO_2_ inhalation 30 min after the last injection, cerebella removed, snap frozen in liquid nitrogen and stored at −80°C.

### Adhesion assays

Adhesion assays were performed in 24-well plates with a confluent layer of activated bEnd.3 cells. T cell blasts or Teff cells were labeled with 5 µM Calcein AM (Molecular Probes, Eugene, OR) and treated as described above. 7×10^5^ pretreated T cell blasts or Teff cells per well were incubated on bEnd.3 cells in RPMI media. After 30 min at 37°C, non-adherent cells were removed by washing with D-PBS. For analysis, 7×10^5^ Calcein AM labeled T cell blasts or Teff cells were used as positive controls. Fluorescence was read in a Victor 3v microplate reader (Perkin Elmer, Waltham, MA) with a fluorescein filter set. The percentage of labeled cells resistant to detachment was calculated as total fluorescence of well divided by fluorescence of 7×10^5^ Calcein AM labeled cells.

### Laser-capture microdissection (LCM)

7 µm cryosections of frozen cerebella were fixed in acetone and rapidly stained according to established protocols [52]. Primary rat anti-CD31 mAb (Pharmingen, San Diego, CA), in conjunction with a biotinylated anti-rat IgG secondary Ab, avidin:biotinylated peroxidase complex, and DAB as an enzyme substrate (all Vector Labs, Burlingame, CA), were used for brain vascular endothelial cell detection and Alexa Fluor 594 conjugated anti-GFAP mAb (Molecular Probes) for astrocyte detection. Selective capture of microvascular endothelial cells or astrocytes was performed using a Pixcell II LCM system (Molecular Devices, Sunnyvale, CA). 500 captures of either CD31^+^ or GFAP^+^ material were taken from a single slide. Cell captures from three slides were pooled and reversely transcribed into cDNA for a total of 1500 captures per cDNA. Three different cDNAs were separately analyzed from each animal. Two animals were used per treatment group. Microphotographs were taken on an Olympus IX51 microscope integrated into the LCM instrument.

### Proliferation assays

T cell-depleted splenocytes (Tds) were obtained by negative selection with murine anti-CD4 and anti-CD8 microbeads (Miltenyi Biotec). Isolated CD4^+^CD25^−^ Teff cells (5×10^4^/well) were cultured in 96-well plates (Costar) with irradiated Tds (5×10^4^/well; 2600 rad) in the presence or absence of soluble anti-CD3 mAb (0.7 µg/ml) (R&D Systems, Minneapolis, MN). DP (100 µM), IBMX (300 µM), PICL (1, 0.1 µM), PF-4957325-00 (1, 0.1 µM), or vehicle control (0.1% DMSO in media) were added at 0 h. After 48 h, 2 µCi per well of [^3^H]thymidine (NEN, Waltham, MA) was added and cells were harvested 16 h later using a semiautomated cell harvester. [^3^H]thymidine incorporation was determined using a β-scintillation counter. Cell viability was examined in separate T cell cultures using trypan blue (2.5%) at 64 h of incubation.

### Intracellular cAMP ELISAs

Activated bEnd.3 cells were treated as described above for 20, 45 or 90 min. cAMP levels were determined with a Correlate cAMP ELISA kit (Assay Designs, Ann Arbor, MI) using an ELISA reader (Bio-Rad, Hercules, CA) with the filter set at 405 nm.

### Statistics

Experimental groups were compared by analyzing data with the unpaired *t*-test or one-way ANOVA followed by Bonferroni *t*-test using Sigmastat software (San Jose, CA). Probability levels for statistically significant differences are indicated by the *p-*value in the figure legend and by corresponding asterisks in the figures.

## Supporting Information

Table S1 Genes and DNA sequences of forward and reverse primers used in qRT-PCR.
(0.07 MB DOC)

Figure S1 αL and α4 integrin expression on Teff cells. Isotype control (unfilled histogram) and specific staining (filled histogram) together with gates for αLhi (Ai) and α4hi (Bi) cells are shown. The cell population staining with a mean florescence intensity above 102 (gated on activated lymphocytes) was defined as αLhi or α4hi. Streptavidin-APC was used to detect α4 integrin, and PE α-CD11a (2D7) to detect αL integrin, or IgG2a,κ and IgG2b, κ as isotype controls.
(0.07 MB TIF)

Figure S2 Figure S2. Structure of the PDE8-selective inhibitor PF-4957325-00. The PDE8-selective inhibitor PF-4957325-00 was developed and synthesized by Pfizer Inc.
(0.13 MB TIF)

Figure S3 Suppression of proliferation is not dependent on inhibition of PDE8. Proliferation of purified CD4+CD25- Teff cells in the presence of PDE-selective inhibitors. Teff cells (5×104/well) were cultured on plate-bound anti-CD3 mAb in the presence of PICL (1 or 0.1 µM), PF-4957325-00 (1 or 0.1 µM), or vehicle control. The extent of proliferation was determined by [3H]thymidine incorporation at 64 h, and results are presented as percentage of proliferation normalized to the vehicle condition. Data are the mean + SEM of 1-3 independent experiments performed in triplicate (*p<0.05, **p<0.001; comparisons to vehicle were analyzed using a one-way ANOVA and Bonferroni t-test; comparisons between PICL and PF-4957325-00 were performed using an unpaired t-test).
(0.08 MB TIF)

Figure S4 DP mediated suppression of adhesion is not reversed by CXCL12. Adhesion of T cell blasts to activated bEnd.3 cells in the presence of CXCL12. Activated T cell blasts were incubated for 45 min with DP (100 µM) or vehicle (0.1% DMSO). Separately, activated bEnd.3 endothelial cells were incubated with DP in the presence or absence of CXCL12 (250 ng/ml) for 45 min before T cell blasts were added to the bEnd.3 cells for the adhesion assay. Values are presented as the mean + SEM percentage of T cell blasts resistant to detachment (*p<0.05, **p<0.001; one-way ANOVA and Bonferroni t-test).
(0.05 MB TIF)

## References

[1] Bender AT, Beavo JA (2006). Cyclic nucleotide phosphodiesterases: molecular regulation to clinical use.. Pharmacol Rev.

[2] Conti M, Beavo J (2007). Biochemistry and physiology of cyclic nucleotide phosphodiesterases: essential components in cyclic nucleotide signaling.. Annu Rev Biochem.

[3] Schillace RV, Carr DW (2006). The role of protein kinase A and A-kinase anchoring proteins in modulating T-cell activation: progress and future directions.. Crit Rev Immunol.

[4] Giembycz MA, Corrigan CJ, Seybold J, Newton R, Barnes PJ (1996). Identification of cyclic AMP phosphodiesterases 3, 4 and 7 in human CD4+ and CD8+ T-lymphocytes: role in regulating proliferation and the biosynthesis of interleukin-2.. Br J Pharmacol.

[5] Glavas NA, Ostenson C, Schaefer JB, Vasta V, Beavo JA (2001). T cell activation up-regulates cyclic nucleotide phosphodiesterases 8A1 and 7A3.. Proc Natl Acad Sci U S A.

[6] Li L, Yee C, Beavo JA (1999). CD3- and CD28-dependent induction of PDE7 required for T cell activation.. Science.

[7] Jin S, Richter W, Conti M, Beavo JA, Francis SH, Houslay MD (2007). Insights into the Physiological Functions of PDE4 from Knockout Mice.. Cyclic Nucleotide Phosphodiesterases in Health and Disease.

[8] Jung S, Zielasek J, Kollner G, Donhauser T, Toyka K (1996). Preventive but not therapeutic application of Rolipram ameliorates experimental autoimmune encephalomyelitis in Lewis rats.. J Neuroimmunol.

[9] Peter D, Jin SL, Conti M, Hatzelmann A, Zitt C (2007). Differential expression and function of phosphodiesterase 4 (PDE4) subtypes in human primary CD4+ T cells: predominant role of PDE4D.. J Immunol.

[10] Ekholm D, Hemmer B, Gao G, Vergelli M, Martin R (1997). Differential expression of cyclic nucleotide phosphodiesterase 3 and 4 activities in human T cell clones specific for myelin basic protein.. J Immunol.

[11] Lerner A, Epstein PM (2006). Cyclic nucleotide phosphodiesterases as targets for treatment of haematological malignancies.. Biochem J.

[12] Bourne HR, Lichtenstein LM, Melmon KL, Henney CS, Weinstein Y (1974). Modulation of inflammation and immunity by cyclic AMP.. Science.

[13] Baillie GS, Scott JD, Houslay MD (2005). Compartmentalisation of phosphodiesterases and protein kinase A: opposites attract.. FEBS Lett.

[14] Sitkovsky MV, Ohta A (2005). The ‘danger’ sensors that STOP the immune response: the A2 adenosine receptors?. Trends Immunol.

[15] Sitkovsky MV (2003). Use of the A(2A) adenosine receptor as a physiological immunosuppressor and to engineer inflammation in vivo.. Biochem Pharmacol.

[16] Frohman EM, Monson NL, Lovett-Racke AE, Racke MK (2001). Autonomic regulation of neuroimmunological responses: implications for multiple sclerosis.. J Clin Immunol.

[17] Beavo J, Houslay M, Francis S, Beavo JA, Francis SH, Houslay MD (2007). Cyclic Nucleotide Phosphodiesterase Superfamily.. Cyclic Nucleotide Phosphodiesterases in Health and Disease.

[18] Lugnier C (2006). Cyclic nucleotide phosphodiesterase (PDE) superfamily: a new target for the development of specific therapeutic agents.. Pharmacol Ther.

[19] Sommer N, Martin R, McFarland HF, Quigley L, Cannella B (1997). Therapeutic potential of phosphodiesterase type 4 inhibition in chronic autoimmune demyelinating disease.. J Neuroimmunol.

[20] Moore CS, Earl N, Frenette R, Styhler A, Mancini JA (2006). Peripheral phosphodiesterase 4 inhibition produced by 4-[2-(3,4-Bis-difluoromethoxyphenyl)-2-[4-(1,1,1,3,3,3-hexafluoro-2-hydrox ypropan-2-yl)-phenyl]-ethyl]-3-methylpyridine-1-oxide (L-826,141) prevents experimental autoimmune encephalomyelitis.. J Pharmacol Exp Ther.

[21] Bielekova B, Richert N, Howard T, Packer AN, Blevins G (2009). Treatment with the phosphodiesterase type-4 inhibitor rolipram fails to inhibit blood-brain barrier disruption in multiple sclerosis.. Mult Scler.

[22] Houslay MD, Schafer P, Zhang KY (2005). Keynote review: phosphodiesterase-4 as a therapeutic target.. Drug Discov Today.

[23] Giembycz MA (2008). Can the anti-inflammatory potential of PDE4 inhibitors be realized: guarded optimism or wishful thinking?. Br J Pharmacol.

[24] Spina D (2008). PDE4 inhibitors: current status.. Br J Pharmacol.

[25] Dong H, Osmanova V, Epstein PM, Brocke S (2006). Phosphodiesterase 8 (PDE8) regulates chemotaxis of activated lymphocytes.. Biochem Biophys Res Commun.

[26] Shulman Z, Shinder V, Klein E, Grabovsky V, Yeger O (2009). Lymphocyte crawling and transendothelial migration require chemokine triggering of high-affinity LFA-1 integrin.. Immunity.

[27] Cinamon G, Shinder V, Alon R (2001). Shear forces promote lymphocyte migration across vascular endothelium bearing apical chemokines.. Nat Immunol.

[28] Laudanna C, Campbell JJ, Butcher EC (1997). Elevation of intracellular cAMP inhibits RhoA activation and integrin-dependent leukocyte adhesion induced by chemoattractants.. J Biol Chem.

[29] Lorenowicz MJ, Fernandez-Borja M, Hordijk PL (2007). cAMP signaling in leukocyte transendothelial migration.. Arterioscler Thromb Vasc Biol.

[30] Seybold J, Thomas D, Witzenrath M, Boral S, Hocke AC (2005). Tumor necrosis factor-alpha-dependent expression of phosphodiesterase 2: role in endothelial hyperpermeability.. Blood.

[31] Sanz MJ, Cortijo J, Taha MA, Cerda-Nicolas M, Schatton E (2007). Roflumilast inhibits leukocyte-endothelial cell interactions, expression of adhesion molecules and microvascular permeability.. Br J Pharmacol.

[32] Ben-Sasson SZ, Hu-Li J, Quiel J, Cauchetaux S, Ratner M (2009). IL-1 acts directly on CD4 T cells to enhance their antigen-driven expansion and differentiation.. Proc Natl Acad Sci U S A.

[33] Bartolome RA, Sanz-Rodriguez F, Robledo MM, Hidalgo A, Teixido J (2003). Rapid up-regulation of alpha4 integrin-mediated leukocyte adhesion by transforming growth factor-beta1.. Mol Biol Cell.

[34] Sullivan GW, Lee DD, Ross WG, DiVietro JA, Lappas CM (2004). Activation of A2A adenosine receptors inhibits expression of alpha 4/beta 1 integrin (very late antigen-4) on stimulated human neutrophils.. J Leukoc Biol.

[35] Fisher DA, Smith JF, Pillar JS, St Denis SH, Cheng JB (1998). Isolation and characterization of PDE8A, a novel human cAMP-specific phosphodiesterase.. Biochem Biophys Res Commun.

[36] Soderling SH, Bayuga SJ, Beavo JA (1998). Cloning and characterization of a cAMP-specific cyclic nucleotide phosphodiesterase.. Proc Natl Acad Sci U S A.

[37] Springer TA (1994). Traffic signals for lymphocyte recirculation and leukocyte emigration: the multistep paradigm.. Cell.

[38] Steinman L (2005). Blocking adhesion molecules as therapy for multiple sclerosis: natalizumab.. Nat Rev Drug Discov.

[39] Thompson WJ, Ross CP, Hersh EM, Epstein PM, Strada SJ (1980). Activation of human lymphocyte high affinity cyclic AMP phosphodiesterase by culture with 1-methyl-3-isobutylxanthine.. J Cyclic Nucleotide Res.

[40] Wunder F, Gnoth MJ, Geerts A, Barufe D (2009). A novel PDE2A reporter cell line: characterization of the cellular activity of PDE inhibitors.. Mol Pharm.

[41] Coeugniet E, Bendtzen K, Bendixen G (1976). Leucocyte migration inhibitory activity of concanavalin-A-stimulated human lymphocytes. Modification by dipyridamole, lysine-acetylsalicylate and heparin.. Acta Med Scand.

[42] Kim HH, Liao JK (2008). Translational therapeutics of dipyridamole.. Arterioscler Thromb Vasc Biol.

[43] Layseca-Espinosa E, Baranda L, Alvarado-Sanchez B, Portales-Perez D, Portillo-Salazar H (2003). Rolipram inhibits polarization and migration of human T lymphocytes.. J Invest Dermatol.

[44] Goldfinger LE, Han J, Kiosses WB, Howe AK, Ginsberg MH (2003). Spatial restriction of alpha4 integrin phosphorylation regulates lamellipodial stability and alpha4beta1-dependent cell migration.. J Cell Biol.

[45] Lim CJ, Han J, Yousefi N, Ma Y, Amieux PS (2007). Alpha4 integrins are type I cAMP-dependent protein kinase-anchoring proteins.. Nat Cell Biol.

[46] Chilcoat CD, Sharief Y, Jones SL (2008). Tonic protein kinase A activity maintains inactive beta2 integrins in unstimulated neutrophils by reducing myosin light-chain phosphorylation: role of myosin light-chain kinase and Rho kinase.. J Leukoc Biol.

[47] Bodor J, Bodorova J, Gress RE (2000). Suppression of T cell function: a potential role for transcriptional repressor ICER.. J Leukoc Biol.

[48] Liu F, Lee SK, Adams DJ, Gronowicz GA, Kream BE (2007). CREM deficiency in mice alters the response of bone to intermittent parathyroid hormone treatment.. Bone.

[49] Netherton SJ, Maurice DH (2005). Vascular endothelial cell cyclic nucleotide phosphodiesterases and regulated cell migration: implications in angiogenesis.. Mol Pharmacol.

[50] Ashikaga T, Strada SJ, Thompson WJ (1997). Altered expression of cyclic nucleotide phosphodiesterase isozymes during culture of aortic endothelial cells.. Biochem Pharmacol.

[51] Gavard J, Gutkind JS (2008). VE-cadherin and claudin-5: it takes two to tango.. Nat Cell Biol.

[52] Kinnecom K, Pachter JS (2005). Selective capture of endothelial and perivascular cells from brain microvessels using laser capture microdissection.. Brain Res Brain Res Protoc.

[53] Beavo JA, Brunton LL (2002). Cyclic nucleotide research – still expanding after half a century.. Nat Rev Mol Cell Biol.

[54] Price GM, Chrobak KM, Tien J (2008). Effect of cyclic AMP on barrier function of human lymphatic microvascular tubes.. Microvasc Res.

[55] Weyrich AS, Denis MM, Kuhlmann-Eyre JR, Spencer ED, Dixon DA (2005). Dipyridamole selectively inhibits inflammatory gene expression in platelet-monocyte aggregates.. Circulation.

[56] Hoffmann R, Wilkinson IR, McCallum JF, Engels P, Houslay MD (1998). cAMP-specific phosphodiesterase HSPDE4D3 mutants which mimic activation and changes in rolipram inhibition triggered by protein kinase A phosphorylation of Ser-54: generation of a molecular model.. Biochem J.

[57] Zhuplatov SB, Masaki T, Blumenthal DK, Cheung AK (2006). Mechanism of dipyridamole's action in inhibition of venous and arterial smooth muscle cell proliferation.. Basic Clin Pharmacol Toxicol.

[58] Vasta V, Beavo JA, Francis SH, Houslay MD (2007). cAMP-Phosphodiesterase 8 Family.. Cyclic Nucleotide Phosphodiesterases in Health and Disease.

[59] Eigler A, Greten TF, Sinha B, Haslberger C, Sullivan GW (1997). Endogenous adenosine curtails lipopolysaccharide-stimulated tumour necrosis factor synthesis.. Scand J Immunol.

[60] Nitta T, Hata M, Gotoh S, Seo Y, Sasaki H (2003). Size-selective loosening of the blood-brain barrier in claudin-5-deficient mice.. J Cell Biol.

[61] Abbott NJ, Ronnback L, Hansson E (2006). Astrocyte-endothelial interactions at the blood-brain barrier.. Nat Rev Neurosci.

[62] Brocke S, Piercy C, Steinman L, Weissman IL, Veromaa T (1999). Antibodies to CD44 and integrin alpha4, but not L-selectin, prevent central nervous system inflammation and experimental encephalomyelitis by blocking secondary leukocyte recruitment.. Proc Natl Acad Sci U S A.

[63] Ghosh S, Goldin E, Gordon FH, Malchow HA, Rask-Madsen J (2003). Natalizumab for active Crohn's disease.. N Engl J Med.

[64] Miller DH, Khan OA, Sheremata WA, Blumhardt LD, Rice GP (2003). A controlled trial of natalizumab for relapsing multiple sclerosis.. N Engl J Med.

[65] Polman CH, O'Connor PW, Havrdova E, Hutchinson M, Kappos L (2006). A randomized, placebo-controlled trial of natalizumab for relapsing multiple sclerosis.. N Engl J Med.

[66] Rudick RA, Stuart WH, Calabresi PA, Confavreux C, Galetta SL (2006). Natalizumab plus interferon beta-1a for relapsing multiple sclerosis.. N Engl J Med.

[67] Yednock TA, Cannon C, Fritz LC, Sanchez-Madrid F, Steinman L (1992). Prevention of experimental autoimmune encephalomyelitis by antibodies against alpha 4 beta 1 integrin.. Nature.

[68] Steinman L (1996). Multiple sclerosis: a coordinated immunological attack against myelin in the central nervous system.. Cell.

[69] Ford ML, Onami TM, Sperling AI, Ahmed R, Evavold BD (2003). CD43 modulates severity and onset of experimental autoimmune encephalomyelitis.. J Immunol.

[70] Li M, Ransohoff RM (2008). Multiple roles of chemokine CXCL12 in the central nervous system: a migration from immunology to neurobiology.. Prog Neurobiol.

[71] Ransohoff RM (2007). Natalizumab for multiple sclerosis.. N Engl J Med.

[72] Steinman L (2004). Immune therapy for autoimmune diseases.. Science.

