# Supplementary material for: PDE8 Regulates Rapid Teff Cell Adhesion and Proliferation Independent of ICER
Source: PLoS One. 2010 Aug 9;5(8):e12011. doi: 10.1371/journal.pone.0012011 (PMC2918507; doi:10.1371/journal.pone.0012011)
Supplement: Table S1 — Genes and DNA sequences of forward and reverse primers used in qRT-PCR. (0.07 MB DOC) [file pone.0012011.s001.doc]

**Table S1. Genes and DNA sequences of forward and reverse primers used in qRT-PCR**a)

| **Gene Name** | **GenBank Accession Number** | **Primer Sequence (5’-3’)*^a^*** |
| --- | --- | --- |
| **PDE1A** | NM_016744 | (fwd) ACTGCTGGACACAGAGGATGA |
|  |  | (rvs) CCCCATTTTGCGTGTGAAAG |
| **PDE1B** | NM_008800 | (fwd) CGAGTGCAGCCAGGTAAAGC |
|  |  | (rvs) CAAGAGAGGAGGAGGCAGTCA |
| **PDE2A** | NM_001008548 | (fwd) AAGTGTGAGTGCCAGGCTCTT |
|  |  | (rvs) TTCTGGCTTCCGTGATGATCT |
| **PDE3B** | NM_011055 | (fwd) TGGTTCTGGACAGATTGCTTACA |
|  |  | (rvs) AATGCAGGGATGTTTGAAGATAGG |
| **PDE4B** | NM_019840 | (fwd) ACCTGAGCAACCCCACCAA |
|  |  | (rvs) CCCCTCTCCCGTTCTTTGTC |
| **PDE5A** | NM_153422 | (fwd) TCAAGGATTCCGAGGGAACA |
|  |  | (rvs) TGGTCCCCTTCATCACTATCAAA |
| **PDE7A** | NM_008802 | (fwd) TCAGCAGCAATCTTGATGCAA |
|  |  | (rvs) AGAGGCTGGGCACTTCACAT |
| **PDE8A** | NM_008803 | (fwd) CCTGCAGCATTCCCAAGTC |
|  |  | (rvs) TGCATAAGGTTAGGCAGGTCAA |
| **VCAM-1** | NM_011693.3 | (fwd) GTGACTCCATGGCCCTCACT |
|  |  | (rvs) CGTCCTCACCTTCGCGTTTA |
| **ICAM-1** | NM_011693.3 | (fwd) ACAGCTCCGTACCTTTGCCA |
|  |  | (rvs) CATCCAACGTGCAAGTCACC |
| **CXCL12** | NM_021704 | (fwd) GCTCCTCGACAGATGCCTTG |
|  |  | (rvs) GACCCTGGCACTGAACTGGA |
| **Claudin-5** | NM_013805 | (fwd) GCTCAGAACAGACTACAGGCACTTT |
|  |  | (rvs) GTGCCCCCAGGATCTCAGTA |
| **IFN-γ** | NM_008337 | (fwd) TCCTCCTGCGGCCTAGCT |
|  |  | (rvs) TGGCAGTAACAGCCAGAAACA |
| **TNF-α** | NM_013693 | (fwd) AACTCCAGGCGGTGCCTAT |
|  |  | (rvs) CGATCACCCCGAAGTTCAGT |
| **IL-2** | NM_008366 | (fwd) GCTCGCATCCTGTGTCACAT |
|  |  | (rvs) CTGCTGTGCTTCCGCTGTAG |
| **CD31** | NM_008816.2 | (fwd) TCCAGGTGTGCGAAATGCT |
|  |  | (rvs) TTTTCGGACTGGCAGCTGAT |
| **GFAP** | NM_010277 | (fwd) ACCGCATCACCATTCCTGTAC |
|  |  | (rvs) TGGCCTTCTGACACGGATTT |
| **RPL19** | NM_009078 | (fwd) CCAAGAAGATTGACCGCCAT |
|  |  | (rvs) CAGCTTGTGGATGTGCTCCAT |

***^a)^***All forward (fwd) and reverse (rvs) primers were chosen using Primer Express software from ABI. Amplicon sizes were approximately 100 bp.
