# Supplementary figures and images for: PDE8 Regulates Rapid Teff Cell Adhesion and Proliferation Independent of ICER
Source: PLoS One. 2010 Aug 9;5(8):e12011. doi: 10.1371/journal.pone.0012011 (PMC2918507; doi:10.1371/journal.pone.0012011)

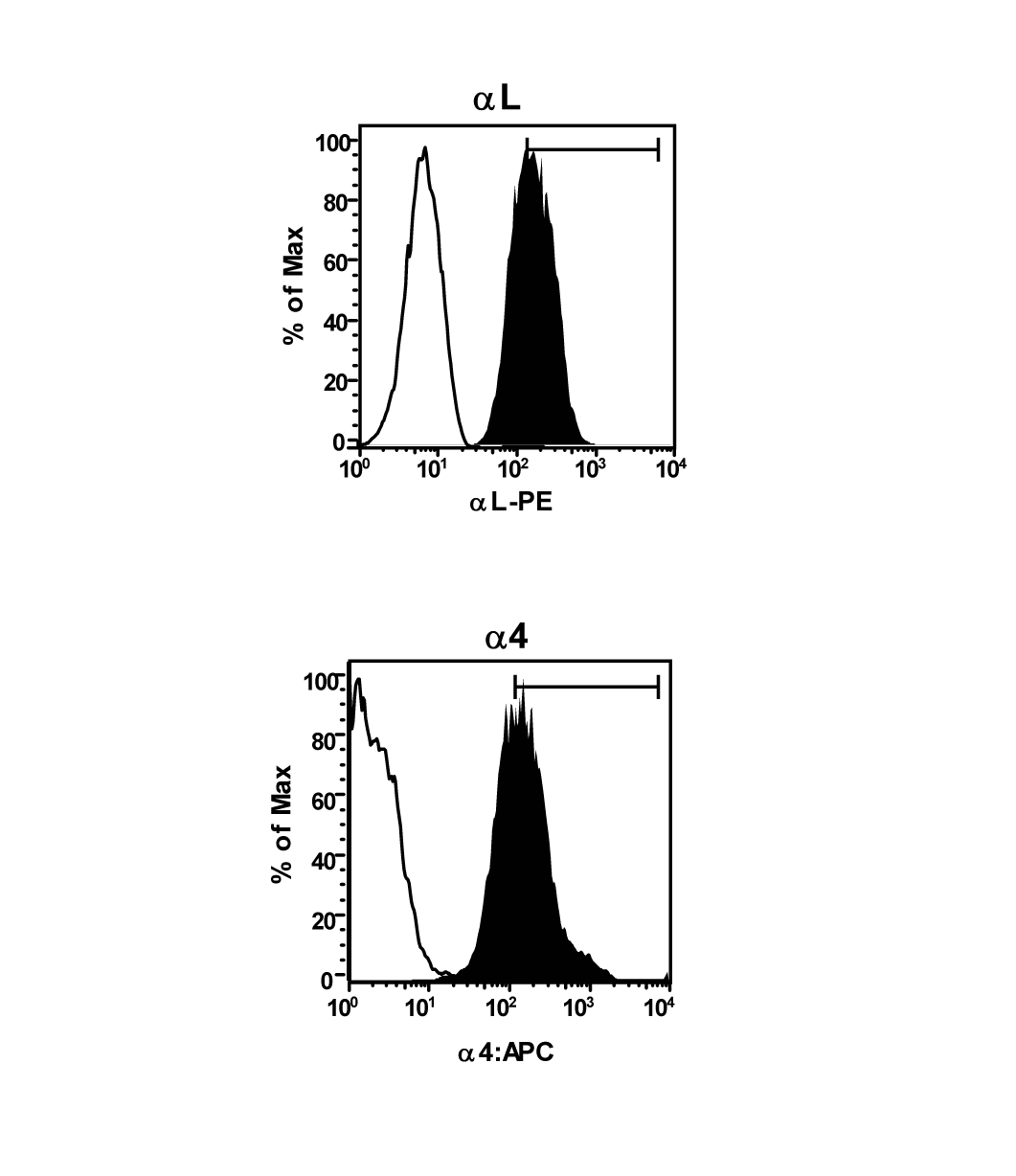

Supplement: Figure S1 — αL and α4 integrin expression on Teff cells. Isotype control (unfilled histogram) and specific staining (filled histogram) together with gates for αLhi (Ai) and α4hi (Bi) cells are shown. The cell population staining with a mean florescence intensity above 102 (gated on activated lymphocytes) was defined as αLhi or α4hi. Streptavidin-APC was used to detect α4 integrin, and PE α-CD11a (2D7) to detect αL integrin, or IgG2a,κ and IgG2b, κ as isotype controls. (0.07 MB TIF) [file pone.0012011.s002.tif]

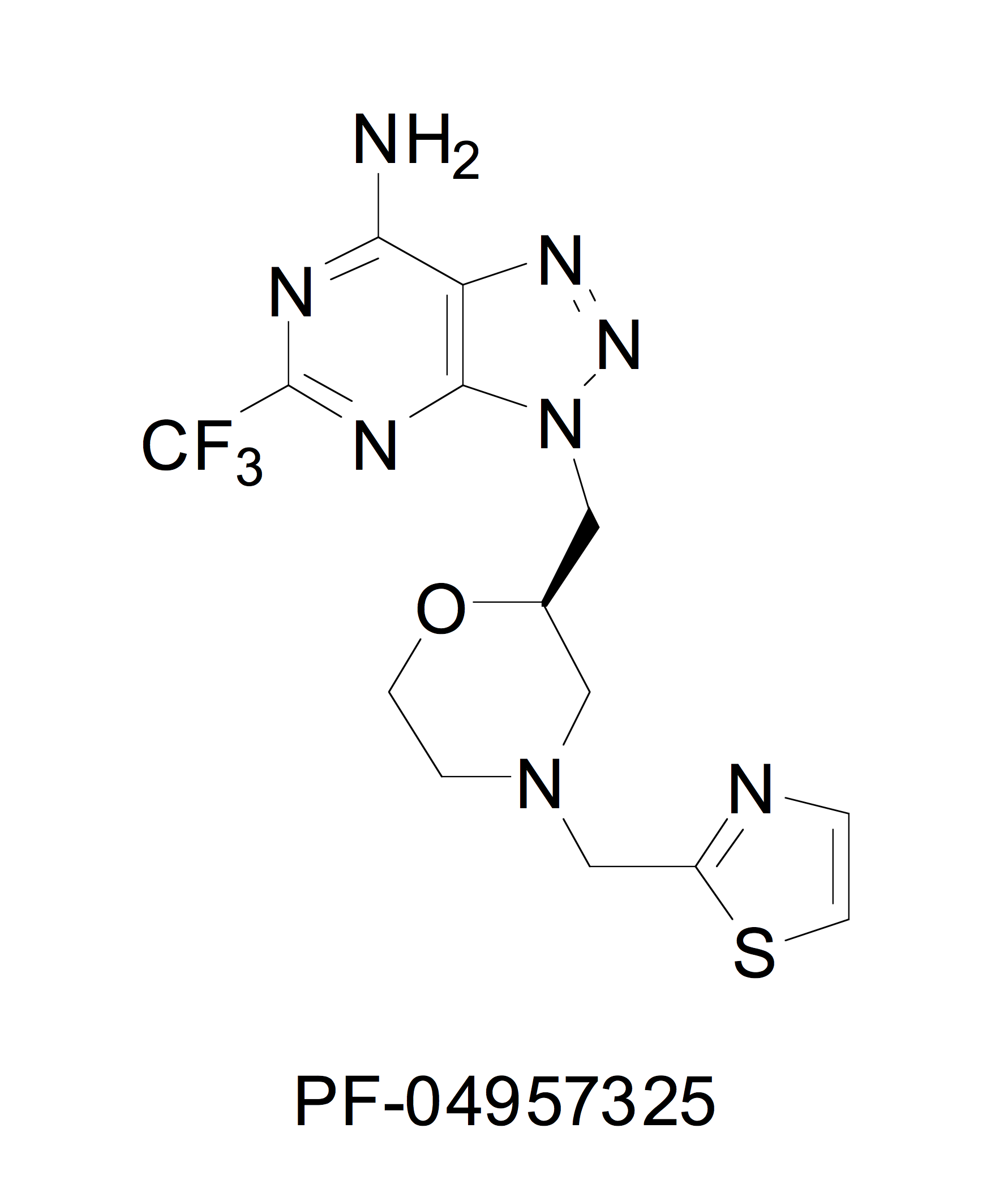

Supplement: Figure S2 — Structure of the PDE8-selective inhibitor PF-4957325-00. The PDE8-selective inhibitor PF-4957325-00 was developed and synthesized by Pfizer Inc. (0.13 MB TIF) [file pone.0012011.s003.tif]

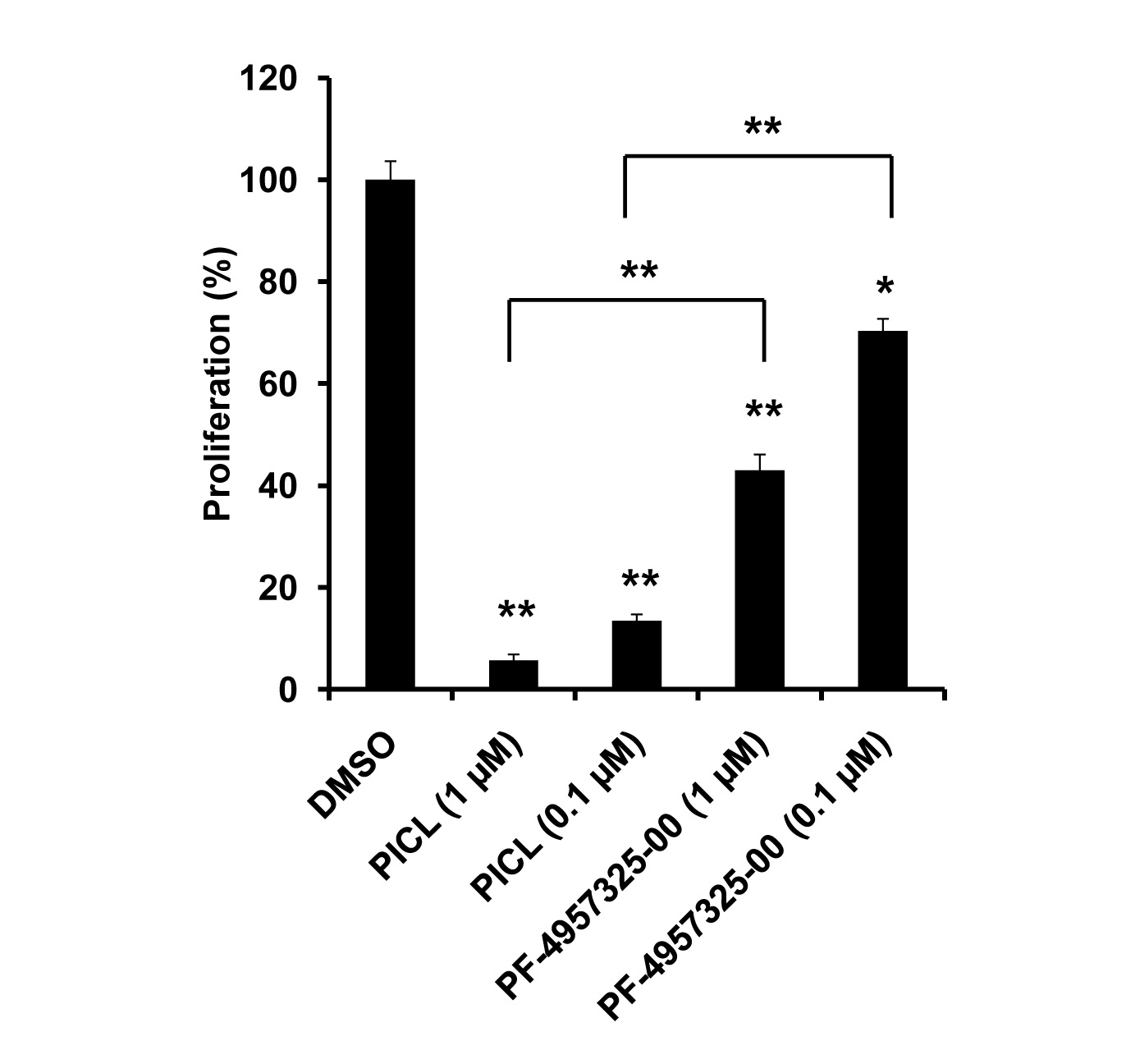

Supplement: Figure S3 — Suppression of proliferation is not dependent on inhibition of PDE8. Proliferation of purified CD4+CD25- Teff cells in the presence of PDE-selective inhibitors. Teff cells (5×10^4^/well) were cultured on plate-bound anti-CD3 mAb in the presence of PICL (1 or 0.1 µM), PF-4957325-00 (1 or 0.1 µM), or vehicle control. The extent of proliferation was determined by [3H]thymidine incorporation at 64 h, and results are presented as percentage of proliferation normalized to the vehicle condition. Data are the mean + SEM of 1-3 independent experiments performed in triplicate (*p<0.05, **p<0.001; comparisons to vehicle were analyzed using a one-way ANOVA and Bonferroni t-test; comparisons between PICL and PF-4957325-00 were performed using an unpaired t-test). (0.08 MB TIF) [file pone.0012011.s004.tif]

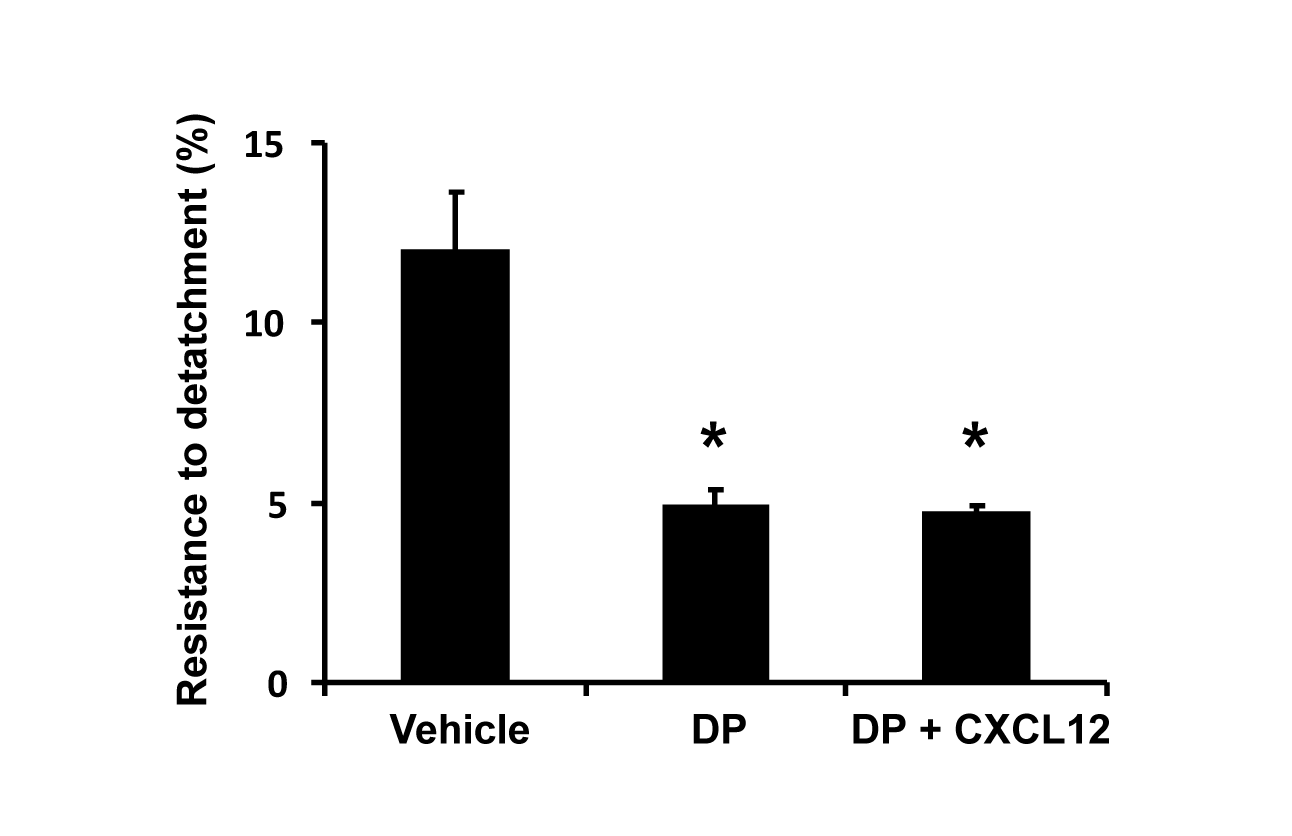

Supplement: Figure S4 — DP mediated suppression of adhesion is not reversed by CXCL12. Adhesion of T cell blasts to activated bEnd.3 cells in the presence of CXCL12. Activated T cell blasts were incubated for 45 min with DP (100 µM) or vehicle (0.1% DMSO). Separately, activated bEnd.3 endothelial cells were incubated with DP in the presence or absence of CXCL12 (250 ng/ml) for 45 min before T cell blasts were added to the bEnd.3 cells for the adhesion assay. Values are presented as the mean + SEM percentage of T cell blasts resistant to detachment (*p<0.05, **p<0.001; one-way ANOVA and Bonferroni t-test). (0.05 MB TIF) [file pone.0012011.s005.tif]
